# Supplementary material for: Microbiome and metabolome features in inflammatory bowel disease via multi-omics integration analyses across cohorts
Source: Nat Commun. 2023 Nov 6;14:7135. doi: 10.1038/s41467-023-42788-0 (PMC10628233; doi:10.1038/s41467-023-42788-0)
Supplement: Supplementary file 1 — Supplementary Information [file 41467_2023_42788_MOESM1_ESM.pdf]

Microbiome and metabolome features in inflammatory bowel disease via multi-omics integration analyses across cohorts

Lijun Ning[1, *], Yi-Lu Zhou[1, *], Han Sun[2, *], Youwei Zhang[3, *], Chaoqin Shen[4, *], Zhenhua Wang[1], Baoqin Xuan[1], Ying Zhao[1], Yanru Ma[1], Yuqing Yan[1], Tianying Tong[1], Xiaowen Huang[1], Muni Hu[1], Xiaoqiang Zhu[1], Jinmei Ding[1], Yue Zhang[1], Zhe Cui[5], Jing-Yuan Fang[1], Haoyan Chen[1], Jie Hong[1]

[1]State Key Laboratory for Oncogenes and Related Genes; Key Laboratory of Gastroenterology & Hepatology, Ministry of Health; Division of Gastroenterology and Hepatology; Shanghai Cancer Institute; Shanghai Institute of Digestive Disease; Renji Hospital, Shanghai Jiao Tong University School of Medicine. 145 Middle Shandong Road, Shanghai 200001, China.

[2]Department of Gastroenterology, Xuzhou Central Hospital, Clinical School of Xuzhou Medical University, Xuzhou, China.

[3]Department of Medical Oncology, Xuzhou Central Hospital, Clinical School of Xuzhou Medical University, Xuzhou, China.

[4]Department of Gastroenterology and Hepatology, Zhongshan Hospital, Fudan University, Shanghai, P.R. China.

[5]Department of Gastrointestinal Surgery, Renji Hospital, Shanghai Jiao Tong University School of Medicine. 145 Middle Shandong Road, Shanghai 200001, China.

These authors contributed equally to this work and share first authorship.

Supplementary Information

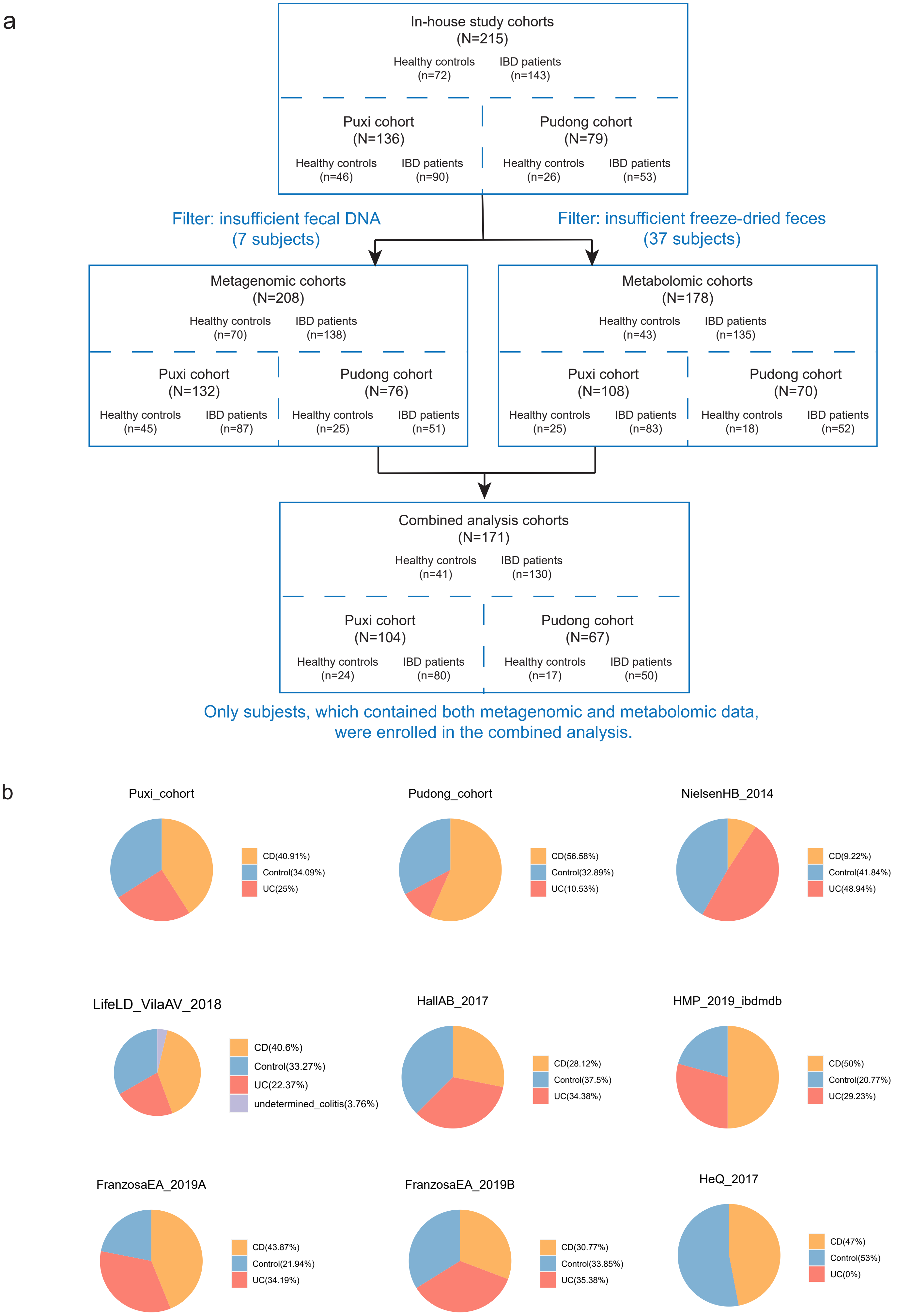
a
In-house study cohorts
(N=215)
Healthy controls (n=72)
IBD patients (n=143)
Puxi cohort
(N=136)
Healthy controls (n=46)
IBD patients (n=90)
Pudong cohort
(N=79)
Healthy controls (n=26)
IBD patients (n=53)
Filter: insufficient fecal DNA
(7 subjects)
Filter: insufficient freeze-dried feces
(37 subjects)
Metagenomic cohorts
(N=208)
Healthy controls (n=70)
IBD patients (n=138)
Puxi cohort
(N=132)
Healthy controls (n=45)
IBD patients (n=87)
Pudong cohort
(N=76)
Healthy controls (n=25)
IBD patients (n=51)
Metabolomic cohorts
(N=178)
Healthy controls (n=43)
IBD patients (n=135)
Puxi cohort
(N=108)
Healthy controls (n=25)
IBD patients (n=83)
Pudong cohort
(N=70)
Healthy controls (n=18)
IBD patients (n=52)
Combined analysis cohorts
(N=171)
Healthy controls (n=41)
IBD patients (n=130)
Puxi cohort
(N=104)
Healthy controls (n=24)
IBD patients (n=80)
Pudong cohort
(N=67)
Healthy controls (n=17)
IBD patients (n=50)
Only subjests, which contained both metagenomic and metabolomic data,
were enrolled in the combined analysis.
b
Puxi_cohort
CD(40.91%)
Control(34.09%)
UC(25%)
Pudong_cohort
CD(56.58%)
Control(32.89%)
UC(10.53%)
NielsenHB_2014
CD(9.22%)
Control(41.84%)
UC(48.94%)
LifeLD_VilaAV_2018
CD(40.6%)
Control(33.27%)
UC(22.37%)
undetermined_colitis(3.76%)
HallAB_2017
CD(28.12%)
Control(37.5%)
UC(34.38%)
HMP_2019_ibdmdb
CD(50%)
Control(20.77%)
UC(29.23%)
FranzosaEA_2019A
CD(43.87%)
Control(21.94%)
UC(34.19%)
FranzosaEA_2019B
CD(30.77%)
Control(33.85%)
UC(35.38%)
HeQ_2017
CD(47%)
Control(53%)
UC(0%)

Supplementary Fig1. Workflow for cross-cohort integration analysis of fecal metagenomics and metabolomics in IBD

a. Recruitment and study design workflow for in-house IBD Renji cohorts. **b.** In this study, a total of 9 metagenomic cohorts from four different regions or countries were analyzed, including 1363 cases. These cohorts were divided into six discovery cohorts and three validation cohorts for further analysis and validation. Pie charts show the percentage composition of disease subtypes in different cohorts.

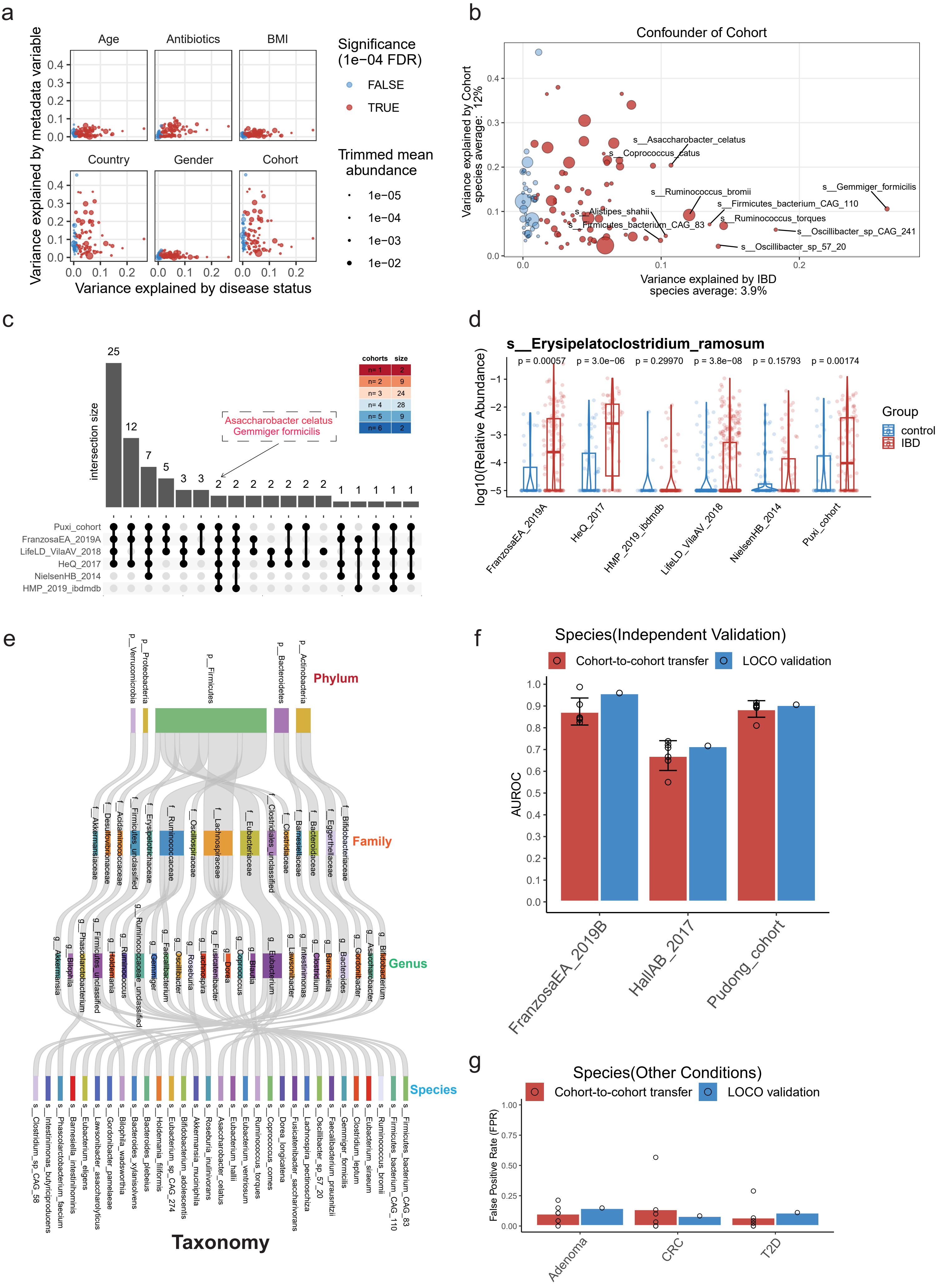

a
Age
Antibiotics
BMI
Country
Gender
Cohort
Variance explained by metadata variable
Variance explained by disease status
Significance (1e−04 FDR)
FALSE
TRUE
Trimmed mean abundance
1e−05
1e−04
1e−03
1e−02
b
Confounder of Cohort
Variance explained by Cohort species average: 12%
Variance explained by IBD species average: 3.9%
s__Asaccharobacter_celatus
s__Coprococcus_catus
s__Ruminococcus_bromii
s__Gemmiger_formicilis
s__Alistipes_shahii
s__Firmicutes_bacterium_CAG_110
s__Ruminococcus_torques
s__Firmicutes_bacterium_CAG_83
s__Oscillibacter_sp_CAG_241
s__Oscillibacter_sp_57_20
c
intersection size
Asaccharobacter celatus
Gemmiger formicilis
cohorts size
n= 1 2
n= 2 9
n= 3 24
n= 4 28
n= 5 9
n= 6 2
Puxi_cohort
FranzosaEA_2019A
LifeLD_VilaAV_2018
HeQ_2017
NielsenHB_2014
HMP_2019_ibdmdb
d
s__Erysipelatoclostridium_ramosum
p = 0.00057
p = 3.0e−06
p = 0.29970
p = 3.8e−08
p = 0.15793
p = 0.00174
log10(Relative Abundance)
Group
control
IBD
FranzosaEA_2019A
HeQ_2017
HMP_2019_ibdmdb
LifeLD_VilaAV_2018
NielsenHB_2014
Puxi_cohort
e
Phylum
Family
Genus
Species
Taxonomy
f
Species(Independent Validation)
Cohort-to-cohort transfer
LOCO validation
AUROC
FranzosaEA_2019B
HallAB_2017
Pudong_cohort
g
Species(Other Conditions)
Cohort-to-cohort transfer
LOCO validation
False Positive Rate (FPR)
Adenoma
CRC
T2D

Supplementary Fig2. Identification of bacterial biomarkers at the species level for diagnosing IBD through cross-cohort demonstration of gut microbiota alterations in IBD patients

a. The analysis aimed to examine the relationship between the variance explained by disease status (IBD versus control) and various putative confounding factors for individual microbial species. The abundance of each microbial species is represented by the size of the corresponding dot on the graph. Among the species analyzed, 74 were found to be significantly different between IBD and control, and are highlighted in red. The analysis considered all available samples to calculate the variance explained by each confounding factor. **b.** Variance explained by disease status (IBD versus control) is plotted against variance explained by cohort effects for individual microbial species with dot size being proportional to abundance. **c.** UpSet plot showing the number of differential bacterial species in each cohort and shared by combinations of datasets. The number above each column represents the size of differential species. The connected dots represent the common differential species across connected cohorts. **d.** The combined boxplot displays the differential relative abundance of *Erysipelatoclostridium ramosum* between the control and IBD. The data is represented using interquartile ranges (IQRs), with the median shown as a black horizontal line, and the whiskers extending to the most extreme points within 1.5 times the IQR. Comparisons were performed using a two-sided Wilcoxon test, and the exact P values are shown. **e.** The Sankey diagram shows a taxonomic chart of 31 feature bacteria, with the majority of the aforementioned features belonging to the phylum Firmicutes. **f.** IBD classification accuracy for independent cohorts is indicated by the bar height for cohort-to-cohort transfer analysis (red) and LOCO (blue) models using species abundance profiles. The error bars indicate the mean ± sd, n = 6. **g.** IBD classification specificity for non-IBD cohorts is indicated by the bar height for cohort-to-cohort transfer analysis (red) and LOCO (blue) models using species abundance profiles as assessed by the FPR.

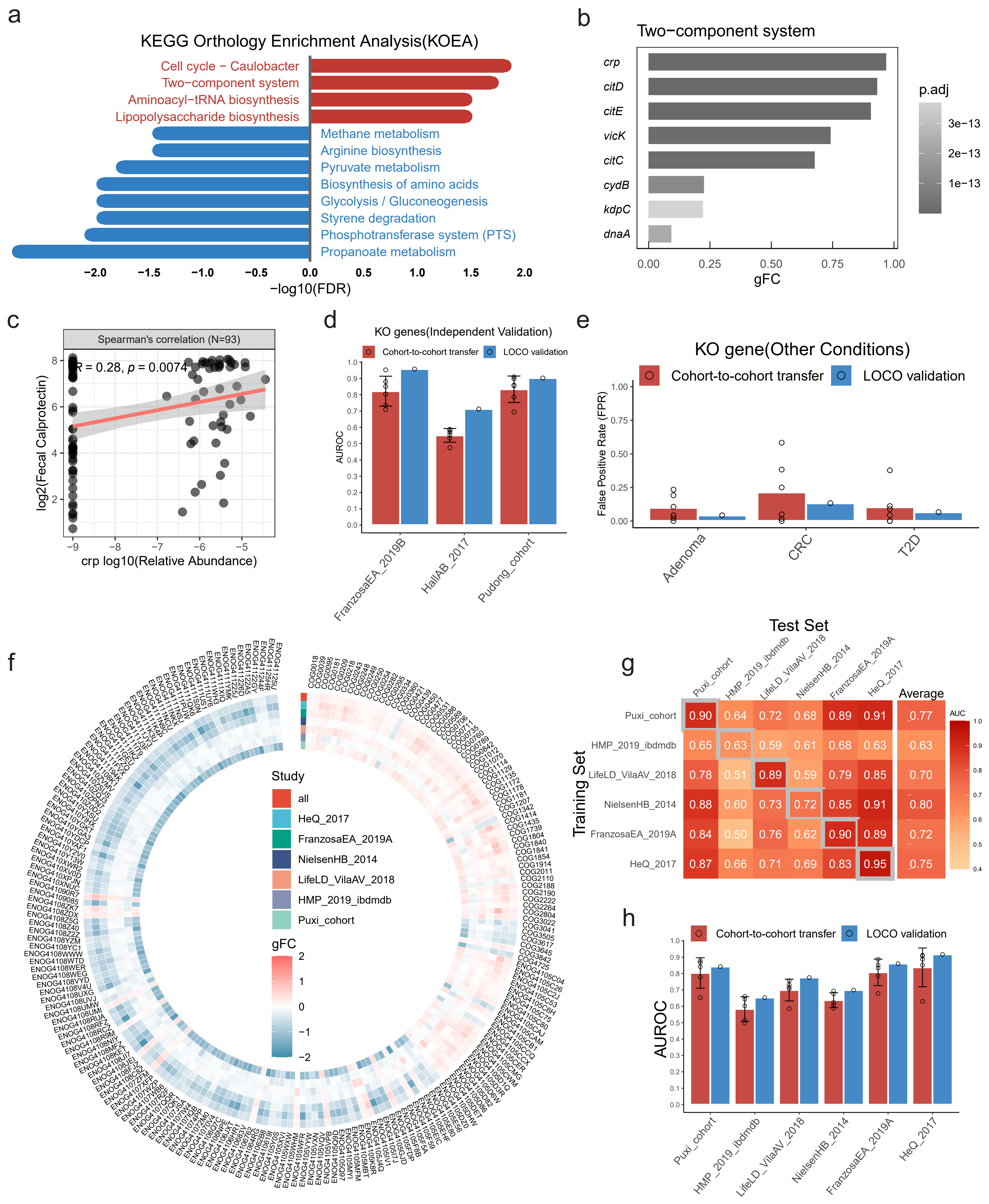
a
KEGG Orthology Enrichment Analysis(KOEA)
Cell cycle − Caulobacter
Two−component system
Aminoacyl−tRNA biosynthesis
Lipopolysaccharide biosynthesis
Methane metabolism
Arginine biosynthesis
Pyruvate metabolism
Biosynthesis of amino acids
Glycolysis / Gluconeogenesis
Styrene degradation
Phosphotransferase system (PTS)
Propanoate metabolism
−2.0 −1.5 −1.0 −0.5 0.0 0.5 1.0 1.5 2.0
−log10(FDR)
b
Two−component system
crp
citD
citE
vicK
citC
cydB
kdpC
dnaA
0.00 0.25 0.50 0.75 1.00
gFC
p.adj
3e−13
2e−13
1e−13
c
Spearman's correlation (N=93)
R = 0.28, p = 0.0074
log2(Fecal Calprotectin)
crp log10(Relative Abundance)
d
KO genes(Independent Validation)
Cohort-to-cohort transfer
LOCO validation
AUROC
FranzosaEA_2019B
HallAB_2017
Pudong_cohort
e
KO gene(Other Conditions)
Cohort-to-cohort transfer
LOCO validation
False Positive Rate (FPR)
Adenoma
CRC
T2D
f
Study
all
HeQ_2017
FranzosaEA_2019A
NielsenHB_2014
LifeLD_VilaAV_2018
HMP_2019_ibdmdb
Puxi_cohort
gFC
2
1
0
−1
−2
g
Test Set
Training Set
Puxi_cohort HMP_2019_ibdmdb LifeLD_VilaAV_2018 NielsenHB_2014 FranzosaEA_2019A HeQ_2017 Average
Puxi_cohort 0.90 0.64 0.72 0.68 0.89 0.91 0.77
HMP_2019_ibdmdb 0.65 0.63 0.59 0.61 0.68 0.63 0.63
LifeLD_VilaAV_2018 0.78 0.51 0.89 0.59 0.79 0.85 0.70
NielsenHB_2014 0.88 0.60 0.73 0.72 0.85 0.91 0.80
FranzosaEA_2019A 0.84 0.50 0.76 0.62 0.90 0.89 0.72
HeQ_2017 0.87 0.66 0.71 0.69 0.83 0.95 0.75
AUC
h
Cohort-to-cohort transfer
LOCO validation
AUROC
Puxi_cohort
HMP_2019_ibdmdb
LifeLD_VilaAV_2018
NielsenHB_2014
FranzosaEA_2019A
HeQ_2017

Supplementary Fig3. Identification of IBD diagnostic markers by demonstrating variations of Kyoto Encyclopedia of Genes and Genomes (KEGG) orthology (KO) across different IBD cohorts

a. The differential bar chart shows the results of KEGG Orthology Enrichment Analysis (KOEA). The red bars represent pathways enriched in IBD, while the blue bars represent pathways enriched in the control group (FDR <0.05, calculated using a two-sided Wilcoxon test with FDR-corrected P values). **b.** The generalized fold change (gFC) and p-value of the 8 KO genes enriched in the Two-Component System pathway (two-sided Wilcoxon test with FDR-corrected P values). **c.** Correlation analysis between the *crp* gene and fecal calprotectin protein (Spearman's correlation, FranzosaEA_2019A cohort, n=93). **d.** IBD classification accuracy for independent cohorts is indicated by the bar height for cohort-to-cohort transfer analysis (red) and LOCO (blue) models using KO genes abundance profiles. The error bars indicate the mean ± sd, n = 6. **e.** IBD classification specificity for non-IBD cohorts is indicated by the bar height for cohort-to-cohort transfer analysis (red) and LOCO (blue) models using KO genes abundance profiles as assessed by the FPR. **f.** The circular complex heatmap displays the 209 most significant EggNOG genes ($P < 1\times10^{-12}$), as calculated using a two-sided Wilcoxon test with FDR-corrected P values in the cross-cohort analysis. The heatmap shows the gFC values of these 209 EggNOG genes, with red indicating enriched and blue indicating depleted in IBD. **g.** IBD classification accuracy resulting from 10-fold cross-validation within each cohort (gray boxes along the diagonal) and cohort-to-cohort model transfer (external validations off-diagonal) as measured by the AUROC for classifiers trained on the EggNOG genes abundance profiles. The last column depicts the average AUROC across the external validations. **h.** Classification accuracy, as evaluated by AUROC on a hold-out cohort, improves if functional data (EggNOG genes) from all other cohorts are combined for training (LOCO validation) relative to models trained on data from a single cohort. The error bars indicate the mean ± sd, n = 5.

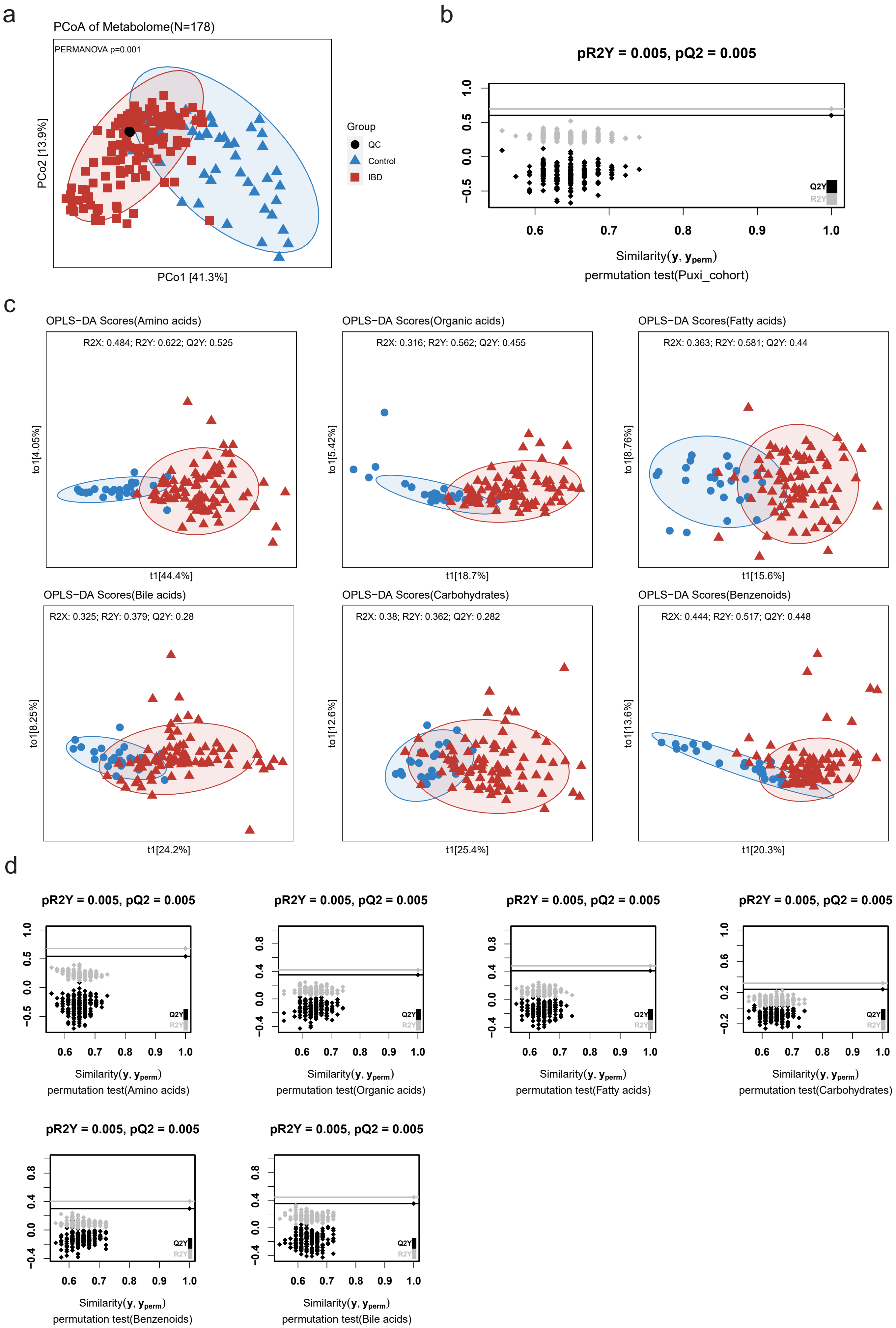
a
PCoA of Metabolome(N=178)
PERMANOVA p=0.001
PCo2 [13.9%]
PCo1 [41.3%]
Group
QC
Control
IBD
b
pR2Y = 0.005, pQ2 = 0.005
Q2Y
R2Y
Similarity(y, yperm)
permutation test(Puxi_cohort)
c
OPLS−DA Scores(Amino acids)
R2X: 0.484; R2Y: 0.622; Q2Y: 0.525
to1[4.05%]
t1[44.4%]
OPLS−DA Scores(Organic acids)
R2X: 0.316; R2Y: 0.562; Q2Y: 0.455
to1[5.42%]
t1[18.7%]
OPLS−DA Scores(Fatty acids)
R2X: 0.363; R2Y: 0.581; Q2Y: 0.44
to1[8.76%]
t1[15.6%]
OPLS−DA Scores(Bile acids)
R2X: 0.325; R2Y: 0.379; Q2Y: 0.28
to1[8.25%]
t1[24.2%]
OPLS−DA Scores(Carbohydrates)
R2X: 0.38; R2Y: 0.362; Q2Y: 0.282
to1[12.6%]
t1[25.4%]
OPLS−DA Scores(Benzenoids)
R2X: 0.444; R2Y: 0.517; Q2Y: 0.448
to1[13.6%]
t1[20.3%]
d
pR2Y = 0.005, pQ2 = 0.005
Similarity(y, yperm)
permutation test(Amino acids)
pR2Y = 0.005, pQ2 = 0.005
Similarity(y, yperm)
permutation test(Organic acids)
pR2Y = 0.005, pQ2 = 0.005
Similarity(y, yperm)
permutation test(Fatty acids)
pR2Y = 0.005, pQ2 = 0.005
Similarity(y, yperm)
permutation test(Carbohydrates)
pR2Y = 0.005, pQ2 = 0.005
Similarity(y, yperm)
permutation test(Benzenoids)
pR2Y = 0.005, pQ2 = 0.005
Similarity(y, yperm)
permutation test(Bile acids)

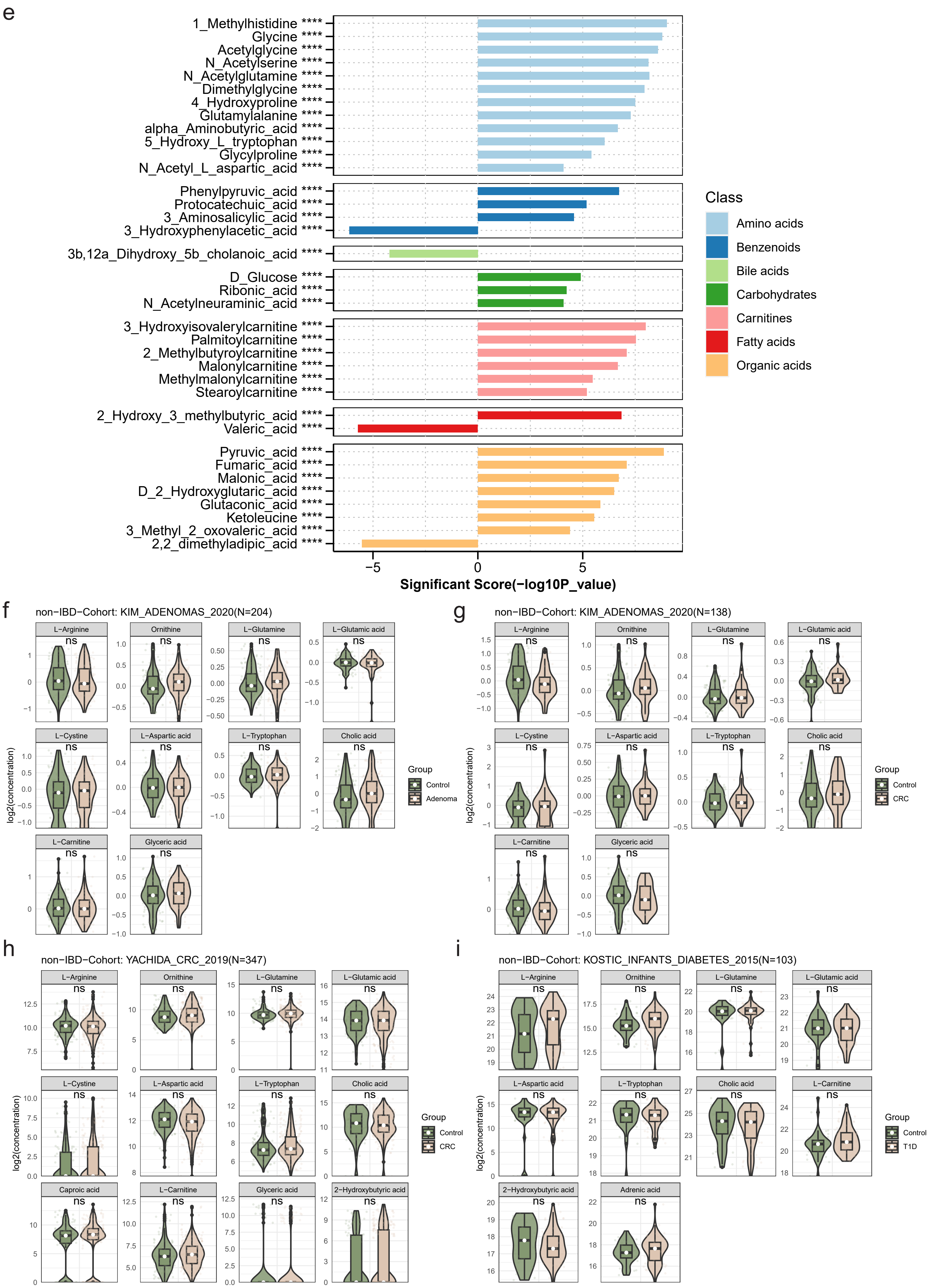
e
1_Methylhistidine ****
Glycine ****
Acetylglycine ****
N_Acetylserine ****
N_Acetylglutamine ****
Dimethylglycine ****
4_Hydroxyproline ****
Glutamylalanine ****
alpha_Aminobutyric_acid ****
5_Hydroxy_L_tryptophan ****
Glycylproline ****
N_Acetyl_L_aspartic_acid ****
Phenylpyruvic_acid ****
Protocatechuic_acid ****
3_Aminosalicylic_acid ****
3_Hydroxyphenylacetic_acid ****
3b,12a_Dihydroxy_5b_cholanoic_acid ****
D_Glucose ****
Ribonic_acid ****
N_Acetylneuraminic_acid ****
3_Hydroxyisovalerylcarnitine ****
Palmitoylcarnitine ****
2_Methylbutyroylcarnitine ****
Malonylcarnitine ****
Methylmalonylcarnitine ****
Stearoylcarnitine ****
2_Hydroxy_3_methylbutyric_acid ****
Valeric_acid ****
Pyruvic_acid ****
Fumaric_acid ****
Malonic_acid ****
D_2_Hydroxyglutaric_acid ****
Glutaconic_acid ****
Ketoleucine ****
3_Methyl_2_oxovaleric_acid ****
2,2_dimethyladipic_acid ****
−5
0
5
Significant Score(−log10P_value)
Class
Amino acids
Benzenoids
Bile acids
Carbohydrates
Carnitines
Fatty acids
Organic acids
f
non−IBD−Cohort: KIM_ADENOMAS_2020(N=204)
L−Arginine
Ornithine
L−Glutamine
L−Glutamic acid
L−Cystine
L−Aspartic acid
L−Tryptophan
Cholic acid
L−Carnitine
Glyceric acid
ns
log2(concentration)
Group
Control
Adenoma
g
non−IBD−Cohort: KIM_ADENOMAS_2020(N=138)
L−Arginine
Ornithine
L−Glutamine
L−Glutamic acid
L−Cystine
L−Aspartic acid
L−Tryptophan
Cholic acid
L−Carnitine
Glyceric acid
ns
log2(concentration)
Group
Control
CRC
h
non−IBD−Cohort: YACHIDA_CRC_2019(N=347)
L−Arginine
Ornithine
L−Glutamine
L−Glutamic acid
L−Cystine
L−Aspartic acid
L−Tryptophan
Cholic acid
Caproic acid
L−Carnitine
Glyceric acid
2−Hydroxybutyric acid
ns
log2(concentration)
Group
Control
CRC
i
non−IBD−Cohort: KOSTIC_INFANTS_DIABETES_2015(N=103)
L−Arginine
Ornithine
L−Glutamine
L−Glutamic acid
L−Aspartic acid
L−Tryptophan
Cholic acid
L−Carnitine
2−Hydroxybutyric acid
Adrenic acid
ns
log2(concentration)
Group
Control
T1D

Supplementary Fig4. Demonstration of Metabolomic Alterations across Different IBD Cohorts and Utilization of Signature Metabolites for IBD Diagnosis

a. A principal coordinates analysis (PCoA) was performed on individuals from the Puxi cohort (n=108). The analysis showed significant differences in metabolites composition between QC, control and IBD (P = 0.001), as determined by PERMANOVA using Bray-Curtis distance with 999 permutations (two-sided test). **b.** The OPLS-DA model validation involves conducting a permutation test on the Puxi cohort (perml = 200, two-sided test, n=108) , which is a nonparametric test. **c.** The OPLS-DA models illustrate the differences between different classified metabolites between control (n = 25) and IBD (n= 83). The full model's predictive performance is evaluated using the cumulative Q2Y metric: Q2Y ranges from 0 to 1, and the higher the Q2Y, the better the performance. **d.** The OPLS-DA model validation involves conducting a permutation test on the different classified metabolites between control (n = 25) and IBD (n= 83) on the Puxi cohort (perml = 200, two-sided test), which is a nonparametric test. **e.** The bar plot illustrates 36 unique identified differential fecal metabolites in the in-house dataset. Their significance scores calculated using a two-sided Wilcoxon test with FDR-corrected P values (denoted as ****p < 0.0001), positive values indicate enrichment in IBD, while negative values indicate enrichment in the control group. **f-i.** The violin plot illustrates the validation of disease specificity for our feature metabolites across four additional non-IBD metabolomics cohorts, which consist of one adenoma cohort, two CRC cohorts, and one T1D cohort. Their significance scores calculated using a two-sided Wilcoxon test with FDR-corrected P values (denoted as ns p > 0.05). Exact p values are provided in the Source data file.

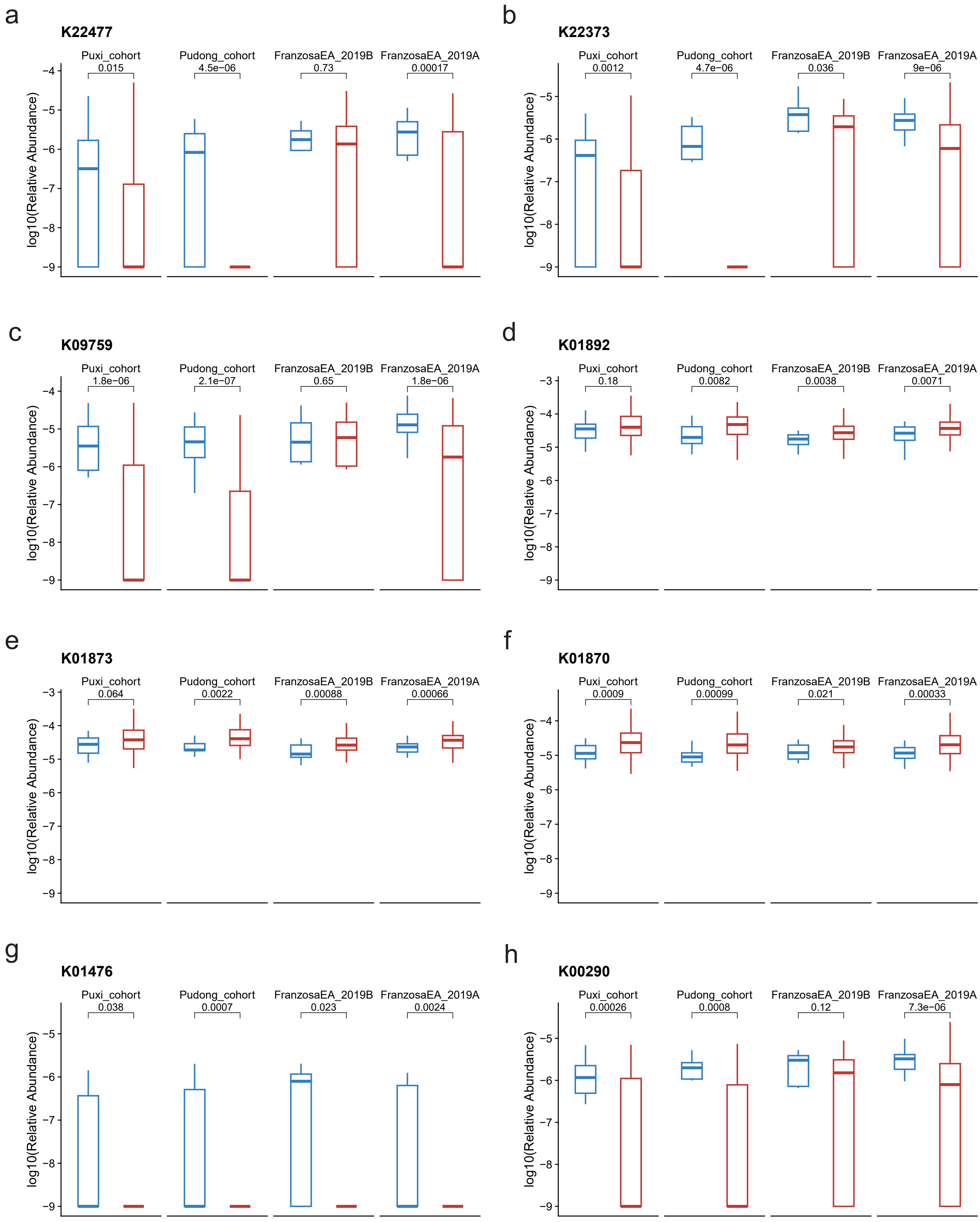
a
K22477
Puxi_cohort
0.015
Pudong_cohort
4.5e−06
FranzosaEA_2019B
0.73
FranzosaEA_2019A
0.00017
log10(Relative Abundance)
b
K22373
Puxi_cohort
0.0012
Pudong_cohort
4.7e−06
FranzosaEA_2019B
0.036
FranzosaEA_2019A
9e−06
log10(Relative Abundance)
c
K09759
Puxi_cohort
1.8e−06
Pudong_cohort
2.1e−07
FranzosaEA_2019B
0.65
FranzosaEA_2019A
1.8e−06
log10(Relative Abundance)
d
K01892
Puxi_cohort
0.18
Pudong_cohort
0.0082
FranzosaEA_2019B
0.0038
FranzosaEA_2019A
0.0071
log10(Relative Abundance)
e
K01873
Puxi_cohort
0.064
Pudong_cohort
0.0022
FranzosaEA_2019B
0.00088
FranzosaEA_2019A
0.00066
log10(Relative Abundance)
f
K01870
Puxi_cohort
0.0009
Pudong_cohort
0.00099
FranzosaEA_2019B
0.021
FranzosaEA_2019A
0.00033
log10(Relative Abundance)
g
K01476
Puxi_cohort
0.038
Pudong_cohort
0.0007
FranzosaEA_2019B
0.023
FranzosaEA_2019A
0.0024
log10(Relative Abundance)
h
K00290
Puxi_cohort
0.00026
Pudong_cohort
0.0008
FranzosaEA_2019B
0.12
FranzosaEA_2019A
7.3e−06
log10(Relative Abundance)

Supplementary Fig5. Construction of a multi-omics biological association map of gut microbiota in IBD

a-h. The box plots display the differences between control and IBD in four distinct cohorts for eight differential KO genes that are associated with metabolism and disease phenotypes. The data is represented using interquartile ranges (IQRs), with the median shown as a black horizontal line, and the whiskers extending to the most extreme points within 1.5 times the IQR. Comparisons were performed using a two-sided Wilcoxon test, and the exact P values are shown. (Puxi_cohort, control=24, IBD=80; Pudong_cohort, control=17, IBD=50; FranzosaEA_2019A, control=34, IBD=121; FranzosaEA_2019B, control=22, IBD=43)

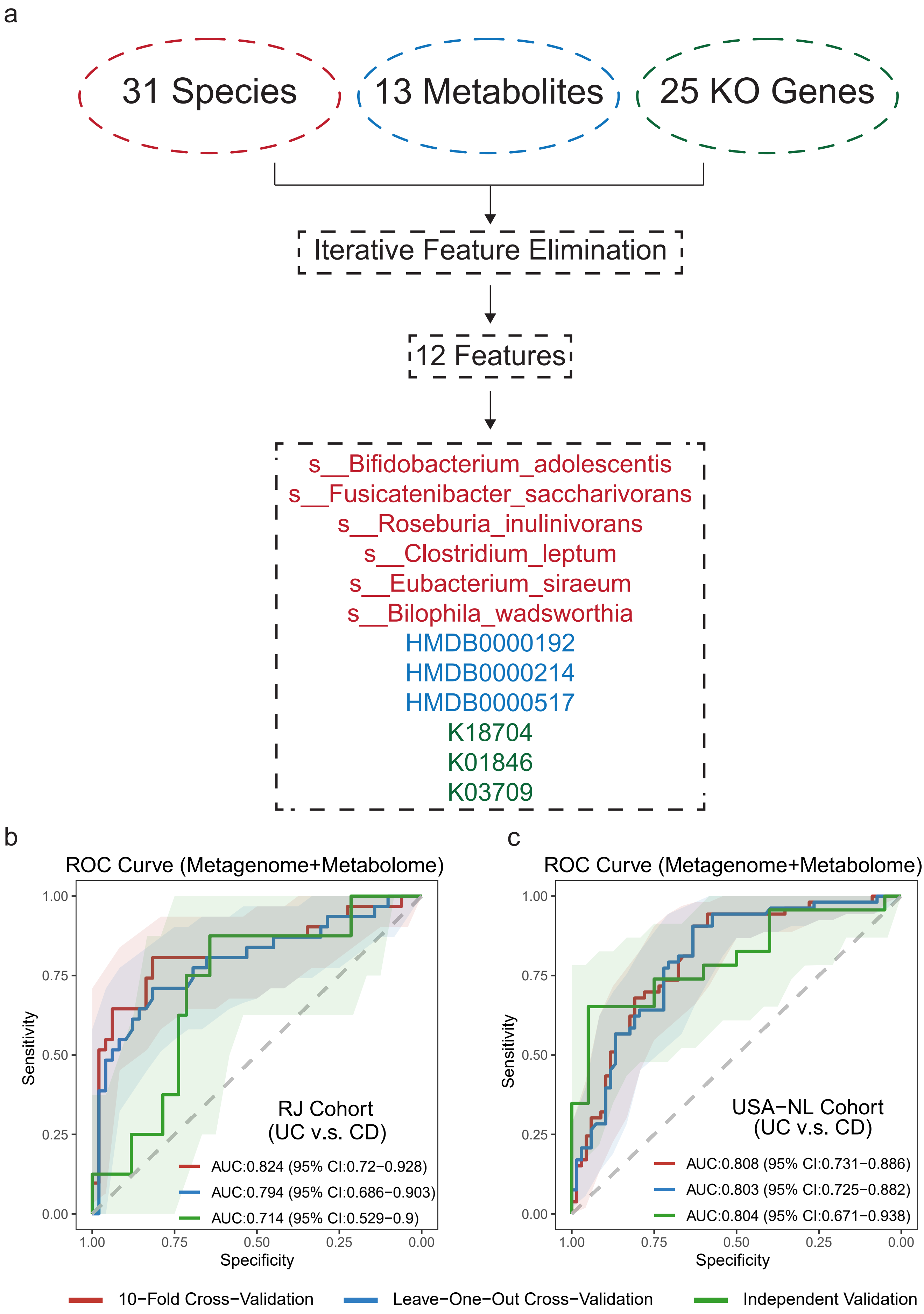
a
31 Species
13 Metabolites
25 KO Genes
Iterative Feature Elimination
12 Features
s__Bifidobacterium_adolescentis
s__Fusicatenibacter_saccharivorans
s__Roseburia_inulinivorans
s__Clostridium_leptum
s__Eubacterium_siraeum
s__Bilophila_wadsworthia
HMDB0000192
HMDB0000214
HMDB0000517
K18704
K01846
K03709
b
ROC Curve (Metagenome+Metabolome)
RJ Cohort
(UC v.s. CD)
AUC:0.824 (95% CI:0.72−0.928)
AUC:0.794 (95% CI:0.686−0.903)
AUC:0.714 (95% CI:0.529−0.9)
Sensitivity
Specificity
c
ROC Curve (Metagenome+Metabolome)
USA−NL Cohort
(UC v.s. CD)
AUC:0.808 (95% CI:0.731−0.886)
AUC:0.803 (95% CI:0.725−0.882)
AUC:0.804 (95% CI:0.671−0.938)
Sensitivity
Specificity
10−Fold Cross−Validation
Leave−One−Out Cross−Validation
Independent Validation

Supplementary Fig6. Identification of multi-omics biomarkers for distinguishing subtypes of IBD

a. Flowchart for identifying 12 features to distinguish between UC and CD. **b-c.** We trained RF classifiers on multi-omics panel to identify IBD subtypes. Training/testing was carried out within the Puxi cohort cohort using 10-fold cross-validation (red) and leave one out cross-validation (LOOCV) (blue), in addition to models trained on the full Puxi cohort, and then tested (validated) on the independent validation (green) in Pudong cohort. Training/testing was carried out within the FranzosaEA_2019A cohort cohort using 10-fold cross-validation (red) and leave one out cross-validation (LOOCV) (blue), in addition to models trained on the full FranzosaEA_2019A cohort, and then tested (validated) on the independent validation (green) FranzosaEA_2019B cohort. Receiver operating characteristic (ROC) curves depict trade-offs between RF classifer true and false positive rates as classification stringency varies. Shaded areas represent the 95% confidence intervals of the corresponding ROC curves.
