# Supplementary material for: Microbiome and metabolome features in inflammatory bowel disease via multi-omics integration analyses across cohorts
Source: Nat Commun. 2023 Nov 6;14:7135. doi: 10.1038/s41467-023-42788-0 (PMC10628233; doi:10.1038/s41467-023-42788-0)
Supplement: Supplementary file 4 — Reporting-summary [file 41467_2023_42788_MOESM4_ESM.pdf]

nature portfolio

Corresponding author(s): Jie Hong, Haoyan Chen

Last updated by author(s): Oct 12, 2023

# Reporting Summary

Nature Portfolio wishes to improve the reproducibility of the work that we publish. This form provides structure for consistency and transparency in reporting. For further information on Nature Portfolio policies, see our Editorial Policies and the Editorial Policy Checklist.

## Statistics

For all statistical analyses, confirm that the following items are present in the figure legend, table legend, main text, or Methods section.

| n/a | Confirmed | |
|---|---|---|
| ☐ | ☒ | The exact sample size (*n*) for each experimental group/condition, given as a discrete number and unit of measurement |
| ☐ | ☒ | A statement on whether measurements were taken from distinct samples or whether the same sample was measured repeatedly |
| ☐ | ☒ | The statistical test(s) used AND whether they are one- or two-sided<br>*Only common tests should be described solely by name; describe more complex techniques in the Methods section.* |
| ☐ | ☒ | A description of all covariates tested |
| ☐ | ☒ | A description of any assumptions or corrections, such as tests of normality and adjustment for multiple comparisons |
| ☐ | ☒ | A full description of the statistical parameters including central tendency (e.g. means) or other basic estimates (e.g. regression coefficient) AND variation (e.g. standard deviation) or associated estimates of uncertainty (e.g. confidence intervals) |
| ☐ | ☒ | For null hypothesis testing, the test statistic (e.g. *F*, *t*, *r*) with confidence intervals, effect sizes, degrees of freedom and *P* value noted<br>*Give P values as exact values whenever suitable.* |
| ☒ | ☐ | For Bayesian analysis, information on the choice of priors and Markov chain Monte Carlo settings |
| ☒ | ☐ | For hierarchical and complex designs, identification of the appropriate level for tests and full reporting of outcomes |
| ☐ | ☒ | Estimates of effect sizes (e.g. Cohen's *d*, Pearson's *r*), indicating how they were calculated |

*Our web collection on statistics for biologists contains articles on many of the points above.*

## Software and code

Policy information about availability of computer code

| | |
|---|---|
| Data collection | Data was downloaded manually from public databases. For China-SH cohort, the metagenomics data sequencing was was carried out on the NovaSeq6000 platform (Illumina, USA). Targeted metabolomics profiling was conducted using the Q300 Metabolite Array Kit from Metabo-Profile Biotechnology of China. |
| Data analysis | MetaPhlAn v.3.0 (https://github.com/biobakery/MetaPhlAn)<br>HUMAnN v.3.0 (https://github.com/biobakery/humann)<br>KneadData v0.10.0 (https://github.com/biobakery/kneaddata)<br>curatedMetagenomicData (v3.6.2) R package<br>vegan (v2.5-7) R package<br>SIAMCAT (v.1.14.0) R package<br>caret (v.6.0.90) R package<br>randomForest (v. 4.7.1.1) R package<br>pROC (v.1.18.0) R package<br>ROCR (v.1.0.11) R package |

For manuscripts utilizing custom algorithms or software that are central to the research but not yet described in published literature, software must be made available to editors and reviewers. We strongly encourage code deposition in a community repository (e.g. GitHub). See the Nature Portfolio guidelines for submitting code & software for further information.

## Data

Policy information about availability of data

All manuscripts must include a data availability statement. This statement should provide the following information, where applicable:

- Accession codes, unique identifiers, or web links for publicly available datasets
- A description of any restrictions on data availability
- For clinical datasets or third party data, please ensure that the statement adheres to our policy

The metagenomics data generated in this study have been deposited in the China National Center for Bioinformation database under accession code PRJCA017408 (https://ngdc.cncb.ac.cn/bioproject/browse/PRJCA017408). Additionally, all other sequencing data analyzed in this work are available in public databases, including the curatedMetagenomicData (PRJNA385949, PRJEB1220, PRJNA398089, EGAS00001001704, EGAD00001004194, PRJEB27928, PRJEB1786, PRJEB7774, https://bioconductor.org/packages/curatedMetagenomicData) and the European Nucleotide Archive (PRJNA400072, PRJEB15371, https://www.ebi.ac.uk/). The metabolomics mass spectral raw data generated in this study have been deposited in MetaboLights under accession code MTBLS8713 (www.ebi.ac.uk/metabolights/ MTBLS8713). The metabolomics data from the external cohorts are sourced from the supplementary materials of their respective articles51. The metabolomics data of non-IBD cohorts are sourced from the study by Muller, E. et al64 (https://github.com/ borenstein-lab/microbiome-metabolome-curated-data). The Human Metabolome Database (HMDB) is a freely accessible electronic database that provides comprehensive information about small molecule metabolites found in the human body (https://hmdb.ca/). Source data are provided as a Source Data file.

## Research involving human participants, their data, or biological material

Policy information about studies with human participants or human data. See also policy information about sex, gender (identity/presentation), and sexual orientation and race, ethnicity and racism.

| | |
|---|---|
| Reporting on sex and gender | In this study, we did not have any specific requirements regarding the participants' gender, and the gender of participants was determined based on self-report. All participants provided informed consent prior to their inclusion in the study. The gender of patients in this study was described separately in Supplementary Table 1. |
| Reporting on race, ethnicity, or other socially relevant groupings | The race, ethnicity, or other socially relevant groupings of patients in this study was described separately in Supplementary Table 1. |
| Population characteristics | In the metagenomic cohorts (Fig 2a), a total of 208 participants were enrolled, comprising 138 patients diagnosed with IBD and 70 healthy control subjects, matched for age and gender. Specifically, the Puxi cohort (N=132, control=45, IBD=87) and the Pudong cohort (N=76, control=25, IBD=51) were employed for both model discovery and validation purposes. For the metabolomic cohorts (Fig 4a), a total of 178 participants were included, with 135 individuals diagnosed with IBD and 43 healthy control subjects, carefully matched for age and gender. Among these, the Puxi cohort (N=108, control=25, IBD=83) and the Pudong cohort (N=70, control=18, IBD=52) were utilized for both model discovery and validation phases. In the combined analysis cohorts (Fig 5a), a total of 171 participants were incorporated, consisting of 130 patients with IBD and 41 age- and gender-matched healthy control subjects. Among them, the Puxi cohort (N=104, control=24, IBD=80) and the Pudong cohort (N=67, control=17, IBD=50) were utilized for both model discovery and validation stages. The details of recruitment for the in-house IBD Renji cohorts are shown in Supplementary Fig. 1a. The clinical characteristics of the study participants are shown in Supplementary Tables 1. |
| Recruitment | In this study, we recruited two IBD cohorts from Renji Hospital, Shanghai, including the Puxi and Pudong campuses, for the discovery and validation cohorts, between between January 1, 2019, and December 31, 2022, respectively. We also recruited a group of healthy control subjects who were carefully matched by age and gender across two hospital campuses. It should be noted that all participants who were enrolled provided informed consent. The enrollment was followed the specific inclusion and exclusion criteria, which are provided in the follow.<br><br>The inclusion criteria included: (1) Participants must be aged between 16 and 65 to be eligible. (2) IBD group were patients newly diagnosed with UC or CD by combining clinical symptoms, imaging, endoscopic and pathological appearances, and had not received any treatment the time of enrollment; (3) Control group was a healthy control population that did not have any significant abnormality in colonoscopy; (4) the participants were capable of understanding and completing the questionnaire, and were willing to cooperate in the collection of fecal samples and basic and clinical information. The exclusion criteria include: (1) medication history of antibiotics, probiotics, immunosuppressants, hormone, or non-steroidal anti-inflammatory drugs within three months before enrollment; (2) abdominal surgery history within six months before enrollment; (3) history of cancer, other autoimmune disease excluding IBD, organ transplantation, or other serious digestive diseases; (4) uncontrolled systemic metabolic disorders such as blood pressure, blood glucose, blood lipids within six months before enrollment; (5) severe and uncontrolled gastrointestinal symptoms such as severe gastrointestinal bleeding, severe diarrhea, severe constipation, gastrointestinal obstruction, etc., within six months before enrollment; (6) significant changes in dietary habits, such as the initiation of a vegan diet, etc., within six months before enrollment; (7) inability to cooperate or unwillingness to cooperate with this study. |
| Ethics oversight | The patient cohorts were approved by the ethics committee of Renji Hospital affiliated to the School of Medicine, Shanghai Jiao Tong University, China, the ethical approval number are 2019-qkwkt-001 and 2021-skt-004. All participants provided informed consent prior to their inclusion in the study. |

Note that full information on the approval of the study protocol must also be provided in the manuscript.

# Field-specific reporting

Please select the one below that is the best fit for your research. If you are not sure, read the appropriate sections before making your selection.

☒ Life sciences ☐ Behavioural & social sciences ☐ Ecological, evolutionary & environmental sciences

For a reference copy of the document with all sections, see nature.com/documents/nr-reporting-summary-flat.pdf

# Life sciences study design

All studies must disclose on these points even when the disclosure is negative.

| | |
|---|---|
| Sample size | We did not employ a statistical method to predetermine the sample size because this analysis is based on a comprehensive examination of public data with a sufficient number of samples. A total of 9 metagenomic cohorts from four different regions or countries (N=1363 cases) were included in this study. These cohorts were divided into six discovery cohorts and three validation cohorts. Additionally, we included four metabolomic cohorts (N=398 cases), of which two external cohorts were examined using non-targeted metabolomics, and two in-house cohorts were examined using targeted metabolomics. |
| Data exclusions | To ensure the accuracy of the diagnostic model, we only retained data from the initial sampling, considering that some subjects were sampled at different time points. Moreover, as country or region is a major confounding factor, we only included subjects from the same country in each dataset to minimize confounding effects. Additionally, due to a significant imbalance between normal controls and IBD cases in the LifeLD VilaAV 2018 cohort, we randomly excluded some normal controls to maintain a ratio of 1:2 to 1:3 between normal controls and IBD cases, enhancing the precision of the study. |
| Replication | All internal and external data can be obtained from public databases, and all the bioinformatics tools used are open-source and free. Given the non-normally distributed nature of microbial data, relevant statistical analyses were performed using non-parametric tests, such as the Wilcoxon signed-rank test. |
| Randomization | Not applicable for this observational case-control study. |
| Blinding | Blinding was not possible because statistical analyses depended on information about disease status . |

# Reporting for specific materials, systems and methods

We require information from authors about some types of materials, experimental systems and methods used in many studies. Here, indicate whether each material, system or method listed is relevant to your study. If you are not sure if a list item applies to your research, read the appropriate section before selecting a response.

## Materials & experimental systems

| n/a | Involved in the study | |
|---|---|---|
| ☒ | ☐ | Antibodies |
| ☒ | ☐ | Eukaryotic cell lines |
| ☒ | ☐ | Palaeontology and archaeology |
| ☒ | ☐ | Animals and other organisms |
| ☒ | ☐ | Clinical data |
| ☒ | ☐ | Dual use research of concern |
| ☒ | ☐ | Plants |

## Methods

| n/a | Involved in the study | |
|---|---|---|
| ☒ | ☐ | ChIP-seq |
| ☒ | ☐ | Flow cytometry |
| ☒ | ☐ | MRI-based neuroimaging |
